# Supplementary material for: Predictors of New Dementia Diagnoses in Elderly Individuals: A Retrospective Cohort Study Based on Prefecture-Wide Claims Data in Japan
Source: Int J Environ Res Public Health. 2021 Jan 13;18(2):629. doi: 10.3390/ijerph18020629 (PMC7828475; doi:10.3390/ijerph18020629)
Supplement: Supplementary file 1 [file ijerph-18-00629-s001.pdf]

International Journal of
Environmental Research and Public Health

MDPI

Supplementary Materials

Table S1. Multivariable logistic regression analyses of predictors for new diagnosis of Alzheimer's disease

Predictors	Age- and sex- adjusted model		Multivariable-adjusted model [a]	
	**OR (95%CI)**	**P-value**	**OR (95%CI)**	**P-value**
Age (per 1-year increase)	1.05 (1.04-1.05) [c]	<0.001	1.04 (1.04-1.05)	<0.001
Female	1.24 (1.20-1.28) [d]	<0.001	1.22 (1.18-1.26)	<0.001
Cerebrovascular disease	1.16 (1.12-1.20)	<0.001	1.12 (1.08-1.16)	<0.001
Diabetes mellitus	0.99 (0.96-1.02)	0.486	0.99 (0.95-1.02)	0.462
Ischemic heart disease	1.00 (0.96-1.04)	0.939	0.98 (0.94-1.02)	0.412
Atrial fibrillation	0.93 (0.88-0.98)	0.012	0.93 (0.87-0.98)	0.010
Depression or use of antidepressants at baseline	1.45 (1.38-1.52)	<0.001	1.32 (1.25-1.39)	<0.001
Use of antipsychotics at baseline	1.45 (1.35-1.56)	<0.001	1.21 (1.12-1.31)	<0.001
Use of anxiolytics at baseline	1.12 (1.09-1.16)	<0.001	1.00 (0.96-1.04)	0.919
Use of hypnotics at baseline	1.27 (1.21-1.33)	<0.001	1.15 (1.08-1.22)	<0.001
Use of antihypertensive drugs at baseline	0.95 (0.92-0.98)	0.002	0.92(0.89-0.95)	<0.001
Use of dyslipidemia drugs at baseline	0.97 (0.94-1.00)	0.084	0.96 (0.93-1.00)	0.029
Use of antithrombotics at baseline	1.12 (1.08-1.16)	<0.001	1.07 (1.03-1.12)	<0.001

Abbreviations: OR, odds ratio; CI, confidence interval. [a]Adjusted for age, sex, cardiovascular risk factors, and neurological or psychiatric risk factors. [b]The OR of age was adjusted for sex. [c]The OR of sex was adjusted for age.

Table S2. Multivariable logistic regression analyses of predictors for new diagnosis of vascular dementia

Predictors	Age- and sex- adjusted model		Multivariable-adjusted model [a]	
	OR (95%CI)	P-value	OR (95%CI)	P-value
Age (per 1-year increase)	1.07 (1.05-1.08) [c]	<0.001	1.06 (1.04-1.08)	<0.001
Female	0.81 (0.69-0.96) [d]	0.014	0.84 (0.71-1.00)	0.050
Cerebrovascular disease	2.41 (2.05-2.82)	<0.001	2.20 (1.83-2.65)	<0.001
Diabetes mellitus	1.12 (0.94-1.33)	0.213	1.06 (0.88-1.27)	0.526
Ischemic heart disease	0.94 (0.76-1.17)	0.589	0.84 (0.67-1.05)	0.118
Atrial fibrillation	1.09 (0.82-1.45)	0.534	0.98 (0.73-1.31)	0.894
Depression or use of antidepressants at baseline	1.86 (1.47-2.36)	<0.001	1.33 (1.02-1.74)	0.036
Use of antipsychotics at baseline	2.90 (2.18-3.86)	<0.001	2.25 (1.64-3.07)	<0.001
Use of anxiolytics at baseline	1.22 (1.01-1.46)	0.036	1.00 (0.80-1.25)	0.980
Use of hypnotics at baseline	1.23 (0.94-1.62)	0.126	0.92 (0.66-1.27)	0.607
Use of antihypertensive drugs at baseline	1.17 (0.99-1.40)	0.071	1.02 (0.85-1.23)	0.813
Use of dyslipidemia drugs at baseline	0.82 (0.68-0.99)	0.036	0.73 (0.60-0.88)	0.001
Use of antithrombotics at baseline	1.66 (1.41-1.97)	<0.001	1.19 (0.98-1.44)	0.084

Abbreviations: OR, odds ratio; CI, confidence interval. [a]Adjusted for age, sex, cardiovascular risk factors, and neurological or psychiatric risk factors. [b]The OR of age was adjusted for sex. [c]The OR of sex was adjusted for age.
